# Quality of Life in Frontline Health Workers Working at Selected Government Hospitals of Federal Level in Nepal: An Observational Study

**DOI:** 10.31729/jnma.9187

**Published:** 2025-08-31

**Authors:** Chandra Bahadur Sunar, Srijana Bhattarai, Damaru Prasad Paneru, Asita Elengoe

**Affiliations:** 1 Department of Health Sciences, Lincoln University College, Malaysia; 2 Public Health and Multi-disciplinary Science, Herd International, Lalitpur; 3 Department of Public Health, School of Health and Allied Science, Pokhara University, Pokhara, Nepal; 4 Department of Applied Science, Lincoln University College, Malaysia

**Keywords:** *frontline health workers*, *hospital*, *quality of life*

## Abstract

**Introduction:**

Quality of life is a crucial dimension of overall wellbeing, particularly for frontline health workers whose roles involve high responsibility and exposure to occupational stressors. This study aimed to describe the quality of life and selected related characteristics among frontline health workers in selected federal-level government hospitals in Nepal.

**Methods:**

A descriptive, cross-sectional study was conducted among 460 participants selected through systematic random sampling. Data were collected using the WHOQOL-BREF tool for quality of life, the PHQ-9 for depressive symptoms, the Rosenberg Self-Esteem Scale, and a work stress questionnaire. Ethical approval was obtained from the Nepal Health Research Council.

**Results:**

Out of 400 respondents, 189 (48.25%) of frontline health workers reported average or low quality of life. Depressive symptoms were present in 104 (26%) of respondents, 239 (59.75%) experienced high work-related stress, and 205 (51.25%) respondents reported low self-esteem.

**Conclusions:**

The findings indicate that a considerable proportion of frontline health workers experience lower levels of quality of life, with notable burdens of stress and depressive symptoms. Workplace policies focusing on reasonable working hours, adequate staffing, and supportive environments are needed to promote well-being and job satisfaction.

## INTRODUCTION

Quality of life (QoL) is a multidimensional concept defined by the World Health Organization as an individual’s perception of their position in life within cultural and personal contexts.^1^ It encompasses physical, psychological, social, and environmental well-being, and is particularly relevant for healthcare professionals whose performance directly affects patient care.

Frontline health workers (FHWs) including doctors, nurses, pharmacists, and laboratory staff form the backbone of health systems. Their well-being influences service quality, safety, and system resilience. However, growing evidence highlights significant occupational and psychological challenges. Depression affects up to one-third of healthcare workers in high-income countries, with greater burdens in low- and middle-income nations due to understaffing, poor infrastructure, and limited support.^2^ In Asia, studies consistently report low QoL among health workers, driven by workload and limited opportunities.^3^ In Nepal, nearly half of nurses in a tertiary hospital reported poor QoL, underscoring the need to assess the status of QoL among FHWs in federal hospitals.^4^

## METHODS

A descriptive, facility-based cross-sectional quantitative study design was employed to assess the quality of life (QoL) among frontline health workers. This design was selected to provide a snapshot of the current situation of frontline health workers and describing their various psychosocial and occupational factors and QoL.

The study was conducted in all federal-level hospitals under the Ministry of Health and Population (MoHP) of Nepal. These hospitals serve as referral centers and are critical to the country’s decentralized health system post-federalization. The total study duration was one year, from February 26, 2024, to December 2024. Data collection was carried out over a four-month period, from May 1, 2024, to August 30, 2024.

Ethical approval was obtained from the Institutional Review Committee of the Nepal Health Research Council (Reference No: 1369). Administrative approval for accessing the hospitals was granted by the Ministry of Health and Population. All participants provided written informed consent prior to participation. Confidentiality and anonymity were maintained throughout data collection, storage, and analysis.

The study included frontline health workers employed at federal-level hospitals under the Ministry of Health and Population (MoHP) who were directly involved in patient care and provided consent to participate. Frontline health workers were operationally defined as clinical professionals engaged in direct patient interaction across departments including emergency, inpatient, outpatient, and intensive care units thus excluding administrative staff and trainees. This included doctors, nurses, health assistants, pharmacists, and laboratory technicians holding a fifth-level position or higher. Individuals on long-term leave, resident student doctors, and medical students working in frontline roles were excluded from the study.

The prevalence of 48 % of health worker experiencing poor quality of life was taken as a p for this study.^5^

P=0.48

Q=0.52

Alpha= 0.05

Z = 1.96

D (error) = 0.05

Non-response rate= 20% (0.2)^6^

Confidence interval=95%

Sample size = (z^2^ × p × q/d^2^) × (1+non-response)

  = {(1.96^2^ × 0.48 × 0.52)/0.05^2^} × (1+0.2)^4^

  = 383+76

  =459

A total of 460 questionnaires were distributed to eligible participants, and complete responses were obtained from 400 frontline health workers, resulting in an effective response rate.

A multistage sampling approach was employed. In the first stage, federal-level hospitals were selected using a simple random sampling technique. Of the 23 federal-level hospitals under the MoHP, 12 hospitals (approximately 50%) were selected to ensure representativeness. Each entity was assigned a unique identification number based on the official government list. Random selection was conducted using lottery draw method to select the hospital from the list.

In the second stage, participants from each selected hospital were chosen through systematic random sampling. From each hospital, 38 frontline health workers were selected. A list of eligible frontline health workers was prepared at each hospital, and a sampling interval (k) was calculated by dividing the total number of listed staff by the number of required participants per hospital which is shown in the table 1. To minimize selection bias, each eligible frontline health worker was assigned a unique identification number, a sequential study-specific code. Systematic random sampling was conducted using a fixed interval (k), with the initial starting point determined via a lottery method. This ensured that the selection process was both random and reproducible across all participating hospitals. Selected individuals were then contacted via mobile phone or in person at their workplace to participate in the study.

For quantitative study, questionnaires were collated using various validated tools: WHOQoL BREF questionnaire was used to assess outcome variables.^3^ Total items with the responses in the likert scale were summed to calculate total score, mean was computed from the total score and categorization was done as taking cut off of mean.^5^ The validated tools were further compiled and pretesting was done in 20 percent of sample size to maintain face validity of the study tool and for further modifications and correction and finalization of tools.^6,7,8^

For validity and reliability scores of tools, PHQ-9 tools was used and cutoff was set to > 10 with sensitivity 94% , specificity 80%.^7^ Similarly, WHOQoL BRIEF questionnaire was used with alpha coefficients 91% and psychological domain alpha coefficients 71%.^4^

WHOQoL BREF questionnaire was used to assess outcome variables i.e. quality of life of frontline health workers. The tools used for assessing independent variables in the study were patient health questionnaire to screen depressive symptoms, work related stress were assessed through work stress questionnaire, and Rosenberg self-esteem scale was used to assess self-esteem among front line health workers.^1-3^

Data were coded, entered, and cleaned using Microsoft Excel and analyzed using Stata MP version 13. Descriptive statistics were used to address the study objectives. Continuous variables were scored, and mean values were used for categorization. Frequencies and percentages were computed for all relevant variables.

## RESULTS

A total of 400 respondents participated in the study and were included in the final analysis. The mean age of the respondents was approximately 32.57 years. Out of total respondents, 271 (67.75%) participants were females. Approximately 240 (60%) of the respondents reported earning an annual income of 7 lakhs or less. 221 (55.25%) participants had seven or fewer years of work experience.

Based on the PHQ-9 tool, 104 (26%) of the respondents reported experiencing depressive symptoms in the past two weeks, using a cutoff score of 10. The mean stress level was 29±0.5. 239 (59.75%) of the respondents reported high stress levels. Selfesteem was measured with a mean score of 16±0.37. A total of 195 (48.75%) respondents reported high self-esteem, while 205 (51.25%) respondents reported low self-esteem (Table 2). The mean overall QoL score was 72.00±0.90. 211 (52.75%) participants reported high QoL and 195 (48.25%) had average or low QoL (Table 3).

**Table 1:** Socio-demographic characteristics of the Respondents (n=400).

Variable / Category	n (%)
Age	
≤ 32 years	215(53.75)
> 32 years	185(46.25)
Gender	
Male	129(32.25)
Female	271(67.75)
Designation	
5th-level frontline workers	204(51.00)
Officer level (nursing)	67(16.75)
Medical officer	81(20.25)
Consultant and above	48(12.00)
Qualification	
PCL level	150 (37.50)
Bachelor	171 (42.75)
Masters and above	79 (19.75)
Annual Income	
≤ 7 Lakhs (NPR)	240 (60.00)
> 7 Lakhs (NPR)	160 (40.00)
Years of experience	
≤ 7 years	221 (55.25)
> 7 years	179 (44.75)

PCL= Proficiency Certificate Level, NPR= Nepalese rupee

**Table 2:** Depressive symptoms, stress and self-esteem descriptions of the respondents (n=400).

SN	Variables	Value
1	Depressive symptoms in the last 2 weeks (cut-off point = 10)	
		Present	104 (26.00)
	Absent	296 (74.00)
2	Stress experienced by respondents	29.00 ± 0.50
	Higher stress level	239 (59.75)
	Below average stress	161 (40.25)
3	Self-esteem	16.00 ± 0.37
	High self-esteem	195 (48.75)
	Low self-esteem	205 (51.25)

**Table 3:** Quality of life of frontline health workers (n=400).

SN	Variables	Value
1	Overall quality of life (QoL) of frontline health workers	72.00 ± 0.90
	High QoL	211 (52.75)
	Average and below QoL	189 (48.25)
	Total	400 (100)
2	Domain of quality of life	
	Physical	75.53 ± 0.97
	Psychological	68.00 ± 0.77
	Environmental	34.00 ± 0.57
	Emotional	88.00 ± 1.32

**Table 4:** Quality of life by socio-demographic and others variables (n=400)

S N	Variables	Quality of life (QoL) of frontline health workers		Total
1	Age	High n (%)	Low n (%)	
	Less than or equal to 32	103(48.82)	112(59.26)	215(53.75)
	More than 32	108(51.18)	77(40.74)	185(46.25)
2	Gender			
	Male	75(35.55)	54(28.57)	129(32.25)
	Female	136 (64.45)	135(71.43)	271(67.75)
3	Designation			
	*5th level frontline workers	100 (47.39)	104(55.03)	204(51.00)
	Officer level and above	111(52.61)	85(44.97)	196(49.00)
4	Qualification			
	PCL level	78(36.97)	72(38.10)	150(37.50)
	Bachelor	89(42.18)	82(43.39)	171(42.75)
	Masters and above	44(20.85)	35(18.52)	79.(19.75)
5	Annual Income			
	7 lakhs and below	115(54.50)	125(66.14)	240(60.00)
	Above 7 lakhs	96(45.50)	64(33.86)	160(40.00)
6	Years of experience			
	7 years and below	103(48.82)	118(62.43)	221(55.25)
	Above 7 years	108(51.18)	71(37.57)	179(44.75)
7	Depressive symptoms in the last 2 weeks			
	Present	4(1.90)	100(52.91)	104(26.00)
	Absent	207(98.10)	89(47.09)	296(74.00)
8	Stress experienced by respondents			
	Higher stress level	61(28.91)	178(94.18)	239(59.75)
	Low stress	150(71.09)	11(5.82)	161(40.25)
9	Self esteem			
	High self esteem	183(86.73)	12(6.35)	195(48.75)
	Low self esteem	28(13.27)	177(93.65)	205(51.25)

Out of 400 frontline health workers, 112(59.26%) respondents aged ≤32 years reported low QoL whereas 108(51.18%) health workers above 32 years reported high QoL 108. 136 (64.45%). 136 (64.45%) female health workers reported high QoL. 104 (55.03%) 5th-level frontline workers had low QoL. 89(42.18) bachelor’s-level health workers, 78 (36.97%) of PCL-level, and 35(18.52%) of master’s-level workers reported high QoL. 125 (66.14%) of those earning ≤7 lakhs annually had low QoL, while 96 (45.50%) of those earning >7 lakhs had high QoL. 118 (62.43%) of those with ≤7 years of experience had low QoL and 108(51.18%) with >7 years reported high QoL. 100(52.91%) of those with depressive symptoms reported low QoL, while 207 (98.10%) of those with high QoL had no depressive symptoms.178 (94.18%) of workers with high stress had low QoL, while 150 (71.09%) of those with low stress reported high QoL. 177 (93.65%) of workers with low QoL had low selfesteem, whereas 183 (86.73%) of those with high QoL had high self-esteem (Table 4).

## DISCUSSION

This study provides critical insights into the quality of life and psychosocial well-being of frontline health workers in Nepal’s federal-level hospitals. The findings reveal that while over half (52.75%) report high QoL, a substantial proportion (48.25%) experience average or low QoL, signaling a significant burden of unmet well-being needs.

The assessment of the quality of life (QoL) of frontline workers is a critical area of research, particularly in the context of their unique work environments and the challenges they face. This study aimed to assess the quality of life of frontline workers, focusing on various determinants such as gender, designation, qualifications, and specific stressors related to their work. The mean overall QoL score of 72 is relatively favorable compared to a previous Nepali study among nurses (mean QoL = 60.74), which reported 48% with below-average QoL.^4^

This discrepancy may reflect differences in cadre (nurses vs. broader FHWs), hospital setting, or temporal changes postpandemic. However, the psychological domain score (68) remains a concern, aligning with global trends of elevated stress and emotional exhaustion among health workers. Our findings resonate with studies from Malaysia and South Korea, where healthcare workers reported high levels of stress and depressive symptoms, particularly during and after the pandemic.^7,8^ Similarly, in Ethiopia, over 40% of health professionals reported low QoL, influenced by workload, lack of recognition, and poor working conditions.^9^ A systematic review confirms that contextual factors such as work environment, leadership support, and job autonomy significantly affect QoL across Low- and middle-income countries.^10^ These finding corresponds to the context of systematic review of quality of life among health workers quoting about different contextual factors are substantial to lower quality of life.^12^ The finding contradicts with the study of Pakistan which reports more than 50 percent frontline workers experiencing low quality of life.^13^. These differences may stem from variations in healthcare financing, staffing ratios, and mental health infrastructure, underscoring the role of national policy in shaping worker well-being. ^11^

The current study has highlighted depressive symptoms as an important variable depicting its symptoms in frontline workers in Nepal. This finding aligns with the findings of countries like Korea, Malaysia and Saudi Arab stating high depressive symptoms, burn out syndrome, stressful work situations among frontline health workers.^9,10,14^ The current finding is also similar to the one of the study done among US volunteers depicting high depressive symptoms and another study of Afghanistan.^15,16^

Depressive symptoms, often manifesting as burnout syndrome, emotional exhaustion, and heightened stress from demanding work environments, are consistently identified as significant predictors of low quality of life across various settings, including Korea, Malaysia, and the United States, highlighting their pervasive impact on psychological well-being and overall life satisfaction.

The current finding reveals low self-esteem among health workers in Nepal. One of the study of India quoted that high self-esteem lead to less work stress and low self-esteem leads to high work stress leading to low quality of life among health workers.^17^ The finding also aligns with another study of quality of life. ^18^

This study provided a descriptive data on psychological factors particularly stress, self-esteem, and depressive symptoms were the strongest determinants of quality of life (QoL) among frontline health workers. The overwhelming proportion of low QoL among those with high stress (94.2%) mirrors findings of Nepal and Ethiopia, who reported that occupational stressors and lack of coping resources significantly impaired well-being.^18^ Likewise, 93.7% of participants with low self-esteem had low QoL, consistent with study of India and Thailand, who demonstrated self-esteem’s protective role in maintaining health-related QoL.^17,18^ Depressive symptoms were also strongly associated with poorer QoL, in line with findings of Malaysia and Afghanistan, highlighting mental health as a central determinant.^10,16^ Hypothetically, if regular workplace-based mental health screening and stress management programs were institutionalized, we could expect a measurable rise in QoL scores within six months.

Demographic and occupational factors also played a role. Older workers (>32 years), those with longer work experience (>7 years), higher incomes, and officer-level positions generally had better QoL, echoing trends noted by study conducted in Nepal, who emphasized the role of job stability, remuneration, and professional recognition in sustaining workforce well-being.^2^'^4^ While gender differences were minimal, other studies have suggested that female staff may face greater cumulative stress from workplace and household responsibilities.^13^ Education level showed little direct impact on QoL, similar to Maqsood et al. (2021).^1^ Even without altering structural demographics, targeted interventions such as resilience workshops and mentorship programs could elevate QoL across groups.

Overall, the findings reinforce that while structural and demographic factors contribute to QoL, psychological wellbeing is the most decisive factor. This aligns with global recommendations (WHO, 2022) to integrate mental health services, stress-reduction initiatives, and self-esteem building strategies into workforce policies.^2^ Hypothetically, embedding such programs into national health workforce strategies could lead not only to improved QoL but also to higher retention and better patient care outcomes.

The main limitation of the study is that it was conducted only among frontline workers from federal-level hospitals, excluding provincial hospitals and local-level health workers, which may limit the generalizability of the findings to the broader healthcare system across the country. The descriptive analysis limits the casual inference of the data preventing significant relationship between the variables.

## CONCLUSIONS

The finding from the study revealed that significant portion of the participants reported a low QoL, emphasizing the need for targeted interventions. This study highlights that QoL among frontline workers is affected by various factors, including age, depressive symptoms, and self-esteem. The finding from the study advocates strengthen workplace policies for ensuring reasonable working hours, adequate staffing, and supportive work environments to reduce burnout and enhance job satisfaction.

## Data Availability

The data are available from the corresponding author upon reasonable request.
